# Supplementary material for: Design and Synthesis of Novel Raman Reporters for Bioorthogonal SERS Nanoprobes Engineering
Source: Int J Mol Sci. 2022 May 16;23(10):5573. doi: 10.3390/ijms23105573 (PMC9144405; doi:10.3390/ijms23105573)
Supplement: Supplementary file 1 [file ijms-23-05573-s001.zip › ijms-1727536-supplementary.pdf]

Supporting Information

Design and Synthesis of Novel Raman Reporters for Bioorthogonal SERS Nanoprobes Engineering

Caterina Dallari [*,†], Riccardo Innocenti [†], Elena Lenci, Andrea Trabocchi, Francesco Saverio Pavone and Caterina Credi

Index supplementary material
S1

Experimental procedures and characterization data for compounds **3a-d** and **4a-d**
S2-S4

Characterization data for NPs constructs
S4-S6

^{1}H and ^{13}C NMR spectra for compounds **3a-d** and **4a-d** S6-S15

General procedure (B) for the thiol deprotection: To a stirred solution of benzamide **3** (1 eq) in DCM (5 mL/mmol), a pre-mixed solution of TFA (5 mL/mmol) and TIPS (3.3 eq) was added at room temperature. The solution turns immediately bright yellow and slightly turns back to colorless when the reaction is complete (usually within 1 hour). Then, TFA was evaporated using a stream of nitrogen and re-evaporated under reduced pressure together with diethyl ether. The product was obtained as white solid after precipitation and filtration or after purification by flash chromatography.

Synthesis of 2-(tritylthio)ethan-1-amine (2)

To a solution of triphenylmethanol (2.080 g, 8.00 mmol) in trifluoroacetic acid (4 mL), 2-thioethylamine hydrochloride (**1**) (1.000 g, 8.80 mmol) was added at 0 °C. The resulting mixture was left stirring at this temperature for 1 h, until the color changed from yellow to red. Then, TFA was evaporated using a stream of nitrogen and re-evaporated under reduced pressure together with acetonitrile. The crude compound, dissolved in ethyl acetate, was washed with a 1M solution of NaOH (3 times) and brine, dried over Na_2SO_4, filtered and evaporated under reduced pressure. Compound **2** (2.271 g, 7.12 mmol) was obtained as a white solid in 89% yield, pure enough to be used in the following steps. Spectroscopical data were found to be in agreement with those reported in the literature.[i]

Synthesis of 4-ethynyl-*N*-(2-(tritylthio)ethyl)benzamide (3a)

According to the general procedure (A), compound **3a** (0.652 g, 1.46 mmol) was obtained as a yellow solid in 91% yield using *p*-ethynylbenzoic acid (0.236 g, 1.61 mmol) and 2-(tritylthio)ethan-1-amine **2** (0.616 g, 1.93 mmol) after flash chromatography on silica gel (Hexane/Et_2O 2:1). ^1H NMR (400 MHz, $CDCl_3$) δ 7.65 (d, *J* = 8.2 Hz, 2H), 7.54 (d, *J* = 8.2 Hz, 2H), 7.45 – 7.38 (m, 6H), 7.24 (dt, *J* = 16.0, 7.0 Hz, 9H), 6.22 (br s, 1H), 3.29 (dd, *J* = 12.2, 6.0 Hz, 2H), 3.19 (s, 1H), 2.54 (t, *J* = 6.2 Hz, 2H). ^{13}C NMR (100 MHz, $CDCl_3$) δ 166.3, 144.5, 134.4, 132.2, 129.5, 128.0, 126.9, 126.8, 82.7, 79.5, 66.9, 38.5, 32.1. MS(ESI) *m*/*z* (%): 470.21 [100, $(M + Na)^+$].

Synthesis of 4-(phenylethynyl)-*N*-(2-(tritylthio)ethyl)benzamide (3b)

According to the general procedure (A), 4-bromo-*N*-(2-(tritylthio)ethyl)benzamide **3i** (1.506 g, 3.00 mmol) was obtained as a white solid in 99% yield using p-bromobenzoic acid (0.603 g, 3.00 mmol) and 2-(tritylthio)ethan-1-amine (1.148 g, 3.60 mmol). Compound **3i** (0.502 g, 1.00 mmol) was transferred into a dry sealed microwaved tube, together with $Pd(PPh_3)_2Cl_2$ (0.035 g, 0.10 mmol), CuI (0.038 g, 0.20 mmol). Then, dry DMF (3 ml), TEA (195 μL, 1.40 mmol) and phenylacetylene (120 μL, 1.10 mmol) were

subsequently added. The resulting mixture was left reacting at 70 °C for 16 hours, then it was diluted with diethyl ether, washed with a 10% solution of NH_4OH (3 times), with a 1 M solution of HCl (3 times) and with brine. The organic phase was dried over Na_2SO_4, concentrated and purified by flash chromatography on silica gel (Pet.Et./EtOAc 3:1) to give compound **3b** in 83% yield (0.434 g, 0.83 mmol). ^{1}H NMR (400 MHz, $CDCl_3$) δ 7.69 (d, *J* = 8.0 Hz, 2H), 7.61 – 7.51 (m, 4H), 7.43 (d, *J* = 8.0 Hz, 4H), 7.42 – 7.36 (m, 4H), 7.32 – 7.19 (m, 10H), 6.27 (s, 1H), 3.31 (dd, *J* = 12.0, 6.0 Hz, 2H), 2.55 (t, *J* = 6.1 Hz, 2H). ^{13}C NMR (100 MHz, $CDCl_3$) δ 166.5, 144.6, 133.7, 131.7, 129.6, 128.7, 128.4, 128.0, 127.9, 127.7, 127.0, 126.9, 126.6, 122.6, 91.6, 88.6, 66.9, 38.6, 32.2. MS(ESI) *m*/*z* (%): 546.14 [100, $(M + Na)^+$].

Synthesis of 4-cyano-*N*-(2-(tritylthio)ethyl)benzamide (3c)

According to the general procedure (A), compound **3c** (0.906 g, 2.02 mmol) was obtained as a white solid in 54% yield using *p*-cyanobenzoic acid (0.618 g, 3.73 mmol) and 2-(tritylthio)ethan-1-amine (1.430 g, 4.48 mmol) after precipitation and filtration of the solid. ^{1}H NMR (400 MHz, $CDCl_3$) δ 7.78 (d, *J* = 7.6 Hz, 2H), 7.70 (dd, *J* = 7.8, 0.7 Hz, 2H), 7.44 – 7.39 (m, 6H), 7.30 – 7.18 (m, 9H), 6.37 (s, 1H), 3.28 (dd, *J* = 12.1, 5.9 Hz, 2H), 2.56 (t, *J* = 6.2 Hz, 2H). ^{13}C NMR (100 MHz, $CDCl_3$) δ 165.5, 144.5, 138.3, 132.4, 129.5, 128.0, 127.9, 127.7, 126.9, 118.0, 115.1, 67.0, 38.8, 31.9. MS(ESI) *m*/*z* (%): 471.04 [100, $(M + Na)^+$].

Synthesis of 4-cyano-2-fluoro-*N*-(2-(tritylthio)ethyl)benzamide (3d)

According to the general procedure (A), compound **3d** (0.430 g, 0.92 mmol) was obtained as a white solid in 92% yield using *m*-fluoro-*p*-cyanobenzoic acid (0.165 g, 1.00 mmol) and 2-(tritylthio)ethan-1-amine (0.382 mg, 1.20 mmol) after precipitation and filtration of the solid. ^{1}H NMR (400 MHz, $CDCl_3$) δ 8.14 (t, *J* = 7.8 Hz, 1H), 7.61 – 7.52 (m, 1H), 7.44 (m, 7H), 7.33 – 7.16 (m, 9H), 6.78 (br s, 1H), 3.29 (q, *J* = 6.1 Hz, 2H), 2.54 (t, *J* = 6.3 Hz, 2H). ^{13}C NMR (100 MHz, $CDCl_3$) δ 161.2, 158.5, 144.5, 133.2, 128.5, 125.4, 120.1, 119.8, 116.4, 66.9, 38.9, 31.6. MS(ESI) *m*/*z* (%): 489.00 [100, $(M + Na)^+$].

Synthesis of 4-ethynyl-*N*-(2-mercaptoethyl)benzamide (4a)

According to the general procedure (B), compound **4a** (0.121 g, 0.59 mmol) was obtained in 63% yield starting from compound **3a** (420 mg, 0.94 mmol) after flash chromatography on silica gel (Hexane/Et_2O 1:1). ^1H NMR (400 MHz, $CDCl_3$) δ 7.55 (dd, *J* = 13.8, 8.2 Hz, 2H), 7.41 – 7.25 (m, 2H), 6.37 (br s, 1H), 3.44 (d, *J* = 6.3 Hz, 2H), 2.99 (s, 1H), 2.65 – 2.53 (m, 2H), 1.22 - 1.20 (m, 1H). ^{13}C NMR (100 MHz, $CDCl_3$) δ 164.5, 134.1, 132.3, 126.9, 125.4, 82.7, 82.0, 42.7, 24.6. MS(ESI) *m*/*z* (%): 228.02 [100, $(M + Na)^+$].

Synthesis of 4-(phenylethynyl)-*N*-(2-mercaptoethyl)-benzamide (4b)

According to the general procedure (B), compound **4b** (0.169 g, 0.60 mmol) was obtained in quantitative yield starting from compound **3b** (0.320 g, 0.60 mmol) after flash chromatography on silica gel (Pet.Et./EtOAc 2:1). ^1H NMR (400 MHz, $CDCl_3$) δ 7.77 (d, *J* = 7.4 Hz, 2H), 7.59 (d, *J* = 7.1 Hz, 2H), 7.54 (ddd, *J* = 6.1, 3.0, 1.4 Hz, 2H), 7.36 (dd, *J* = 4.0, 2.5 Hz, 3H), 6.64 (br s, 1H), 3.65 (q, *J* = 6.3 Hz, 2H), 2.80 (dd, *J* = 14.1, 6.4 Hz, 2H), 1.42 (t, *J* = 8.0 Hz, 1H). ^{13}C NMR (100 MHz, $CDCl_3$) δ 166.8, 133.5, 131.8, 131.7, 128.7, 128.5, 128.4, 127.1, 126.7, 126.8, 122.7, 91.9, 88.5, 42.8, 24.7. MS(ESI) *m*/*z* (%): 304.16 [100, $(M + Na)^+$].

Synthesis of 4-cyano-*N*-(2-mercaptoethyl)benzamide (4c)

According to the general procedure (B), compound **4c** (0.350 g, 1.69 mmol) was obtained in 85% yield starting from compound **3c** (906 mg, 2.00 mmol) after flash chromatography on silica gel (Hexane/AcOEt 1:1). ^1H NMR (400 MHz, $CDCl_3$) δ 7.88 (d, *J* = 8.1 Hz, 2H), 7.71 (d, *J* = 8.1 Hz, 2H), 6.91 (s, 1H), 3.62 (dd, *J* = 12.2, 6.1 Hz, 2H), 2.78 (dd, *J* = 13.9, 7.2 Hz, 2H), 1.41 (t, *J* = 8.4 Hz, 1H). ^{13}C NMR (100 MHz, $CDCl_3$) δ 165.9, 138.2, 132.5, 127.7, 118.0, 115.1, 42.9, 24.5. MS(ESI) *m*/*z* (%): 229.51 [100, $(M + Na)^+$].

Synthesis of 4-cyano-2-fluoro-*N*-(2-mercaptoethyl)benzamide (4d)

According to the general procedure (B), compound **4d** (0.043 g, 0.19 mmol) was obtained in 42% yield starting from compound **3d** (0.210 g, 0.45 mmol) after flash chromatography on silica gel (EtPet/AcOEt 2:1). ^{1}H NMR (400 MHz, $CDCl_3$) δ 8.21 (t, *J* = 7.9 Hz, 1H), 7.58 (dd, *J* = 8.1, 1.3 Hz, 1H), 7.46 (d, *J* = 11.0 Hz, 1H), 7.10 (s, 1H), 3.68 (q, *J* = 6.2 Hz, 2H), 2.80 (dt, *J* = 8.4, 6.4 Hz, 2H), 1.43 (t, *J* = 8.5 Hz, 1H). ^{13}C NMR (100 MHz, $CDCl_3$) δ 161.0, 158.5, 133.3, 128.6, 125.3, 119.9, 116.7, 116.6, 43.1, 24.3. MS(ESI) *m/z* (%): 247.23 [100, $(M + Na)^+$].

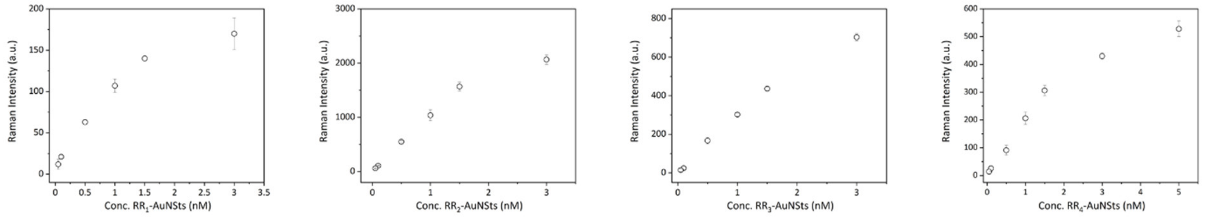

Figure S1. Raman intensity of the characteristic peaks of the four different RR-NP conjugates. (n = 3; error bars represent standard deviation)

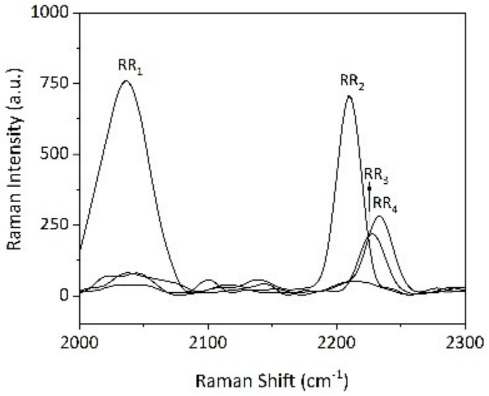

Figure S2. Overlapped Raman-SERS spectra of RR_{1-4}@AuNSts solution at 15nM.

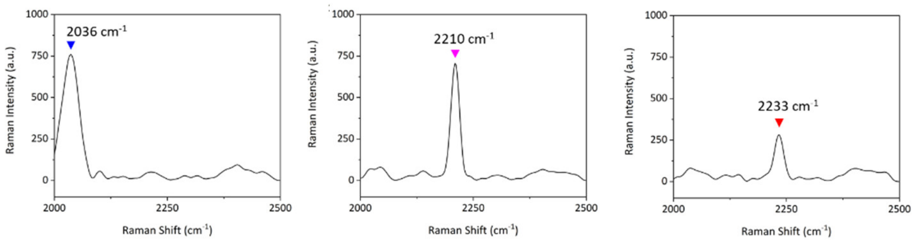

Figure S3. Raman-SERS spectra of RR@AuNSts constructs after antibody conjugation. All spectra are acquired against 15 nM aqueous solution.

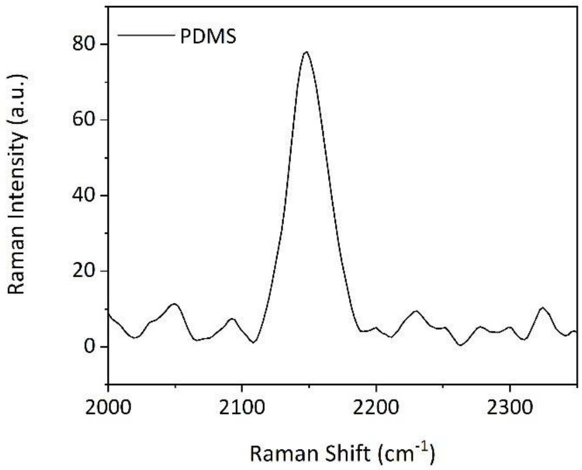

Figure S4. Raman characteristic peak deriving from polymeric substrate.

<table>
<tr><th>Sample
RR@PEG@AuNSts</th><th>Cit-AuNSts</th><th>SH-PEG-COOH</th><th>RR solution</th></tr>
<tr><td>5k</td><td rowspan="4">500 µl @ 2 nM</td><td rowspan="4">500 ul @ 1 mM</td><td>2 µl @ 2.5 mM</td></tr>
<tr><td>10k</td><td>2 µl @ 5 mM</td></tr>
<tr><td>25k</td><td>2 µl @ 12 mM</td></tr>
<tr><td>50k</td><td>2 µl @ 25 mM</td></tr>
</table>

Figure S5. Schematic representation of the optimized experimental conditions for the ligand exchange process employed in the preparation of RR@AuNSts complexes.

Calculation of limit of detection (LOD) and its standard deviation

(1) $LOD = \frac{3*y_{blank}}{m}$

(2) $\Delta LOD = \frac{\Delta y_{blank}}{m} + \frac{y_{blank}\Delta m}{m^2}$

y_{blank}= mean of Raman intensity values from the replicate blank

m= slope of regression curve

Δ= standard deviation

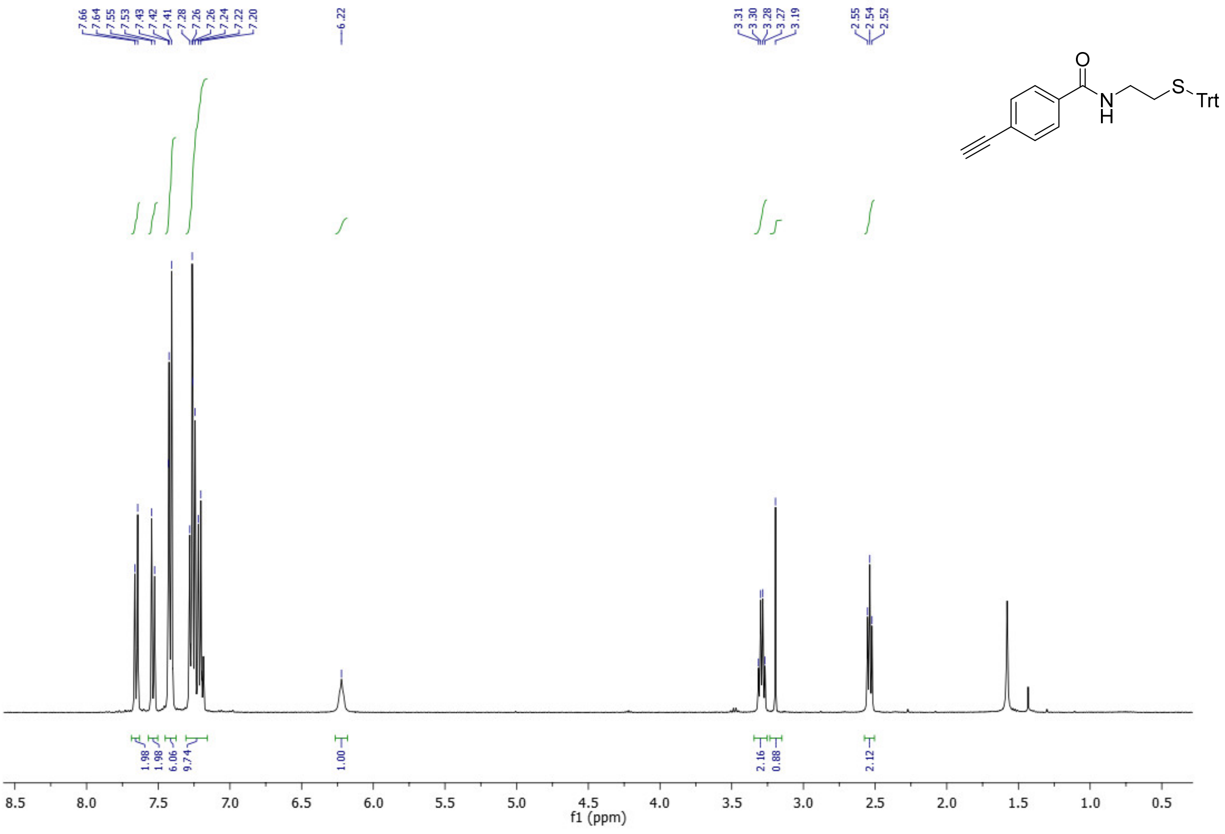

Figure S6. ^{1}H NMR spectrum of compound **3a.**

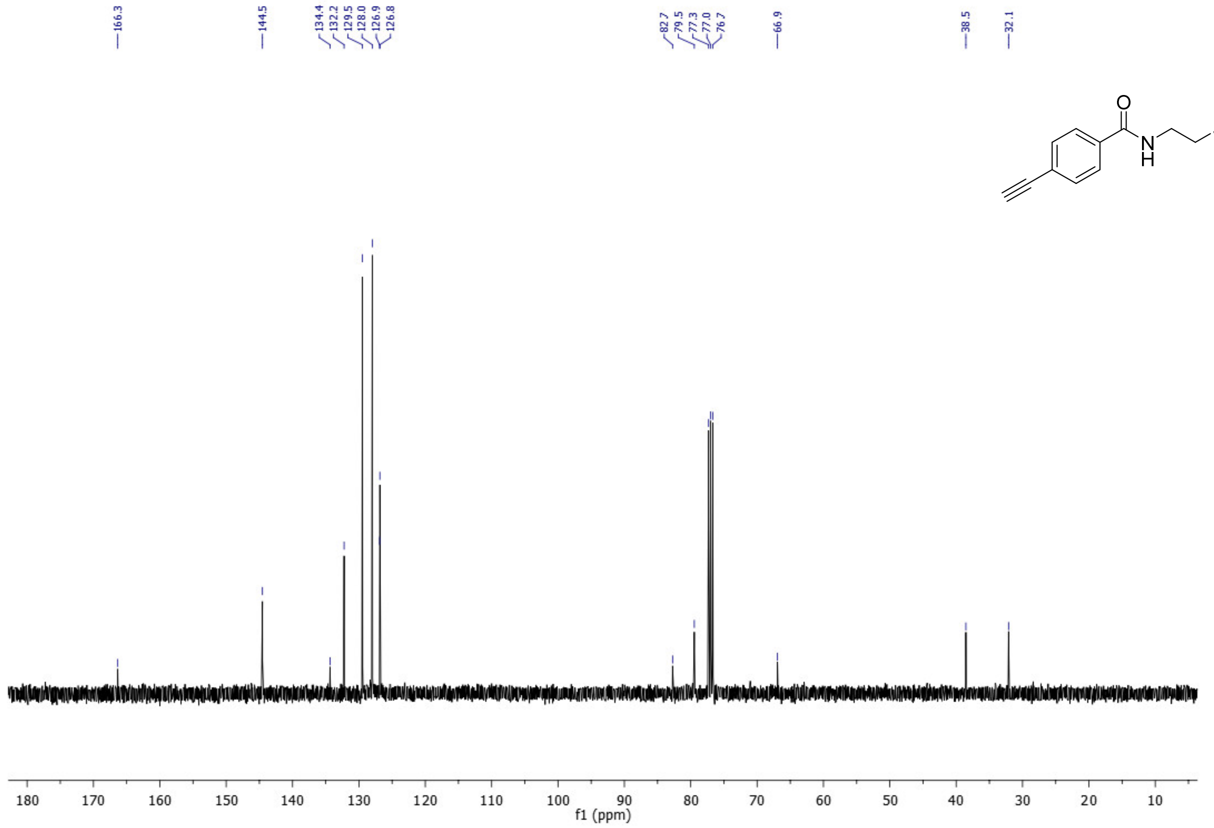

Figure S7. ^{13}C NMR spectrum of compound **3a.**

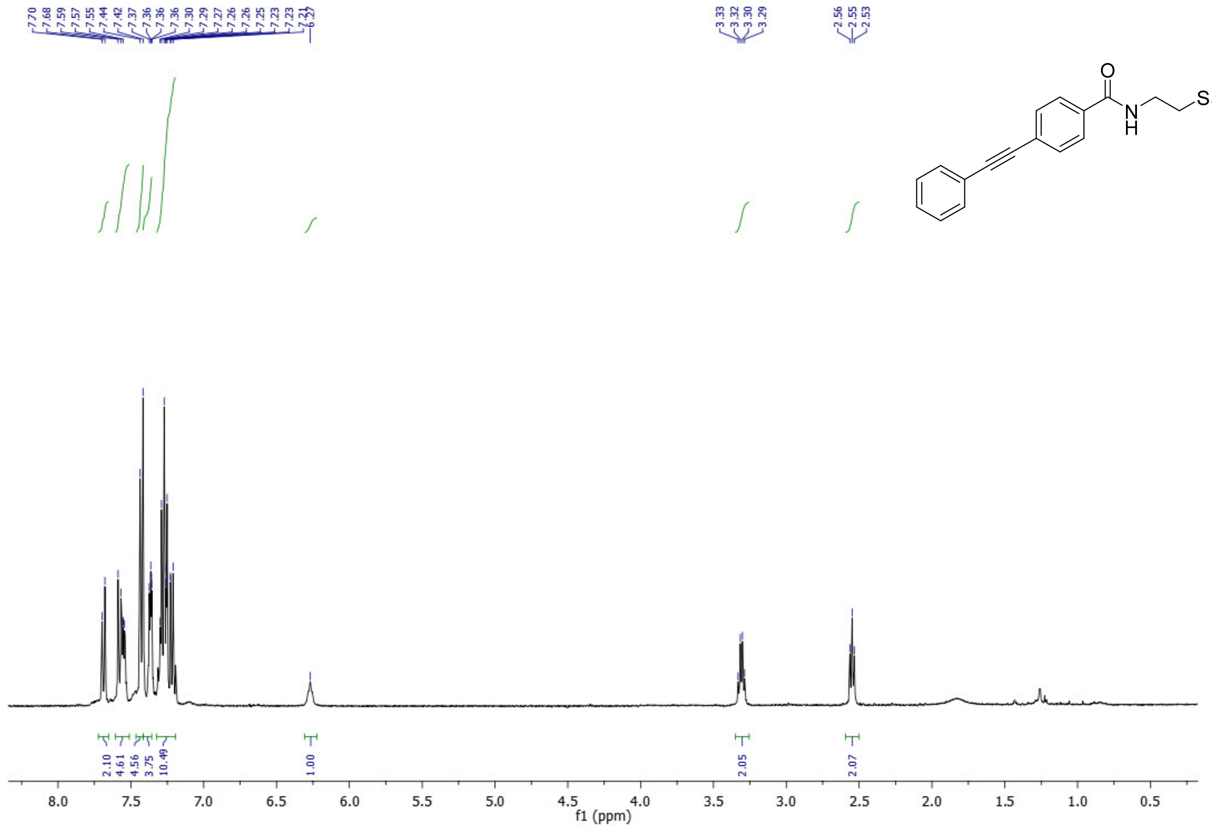

Figure S8. ^{1}H NMR spectrum of compound **3b.**

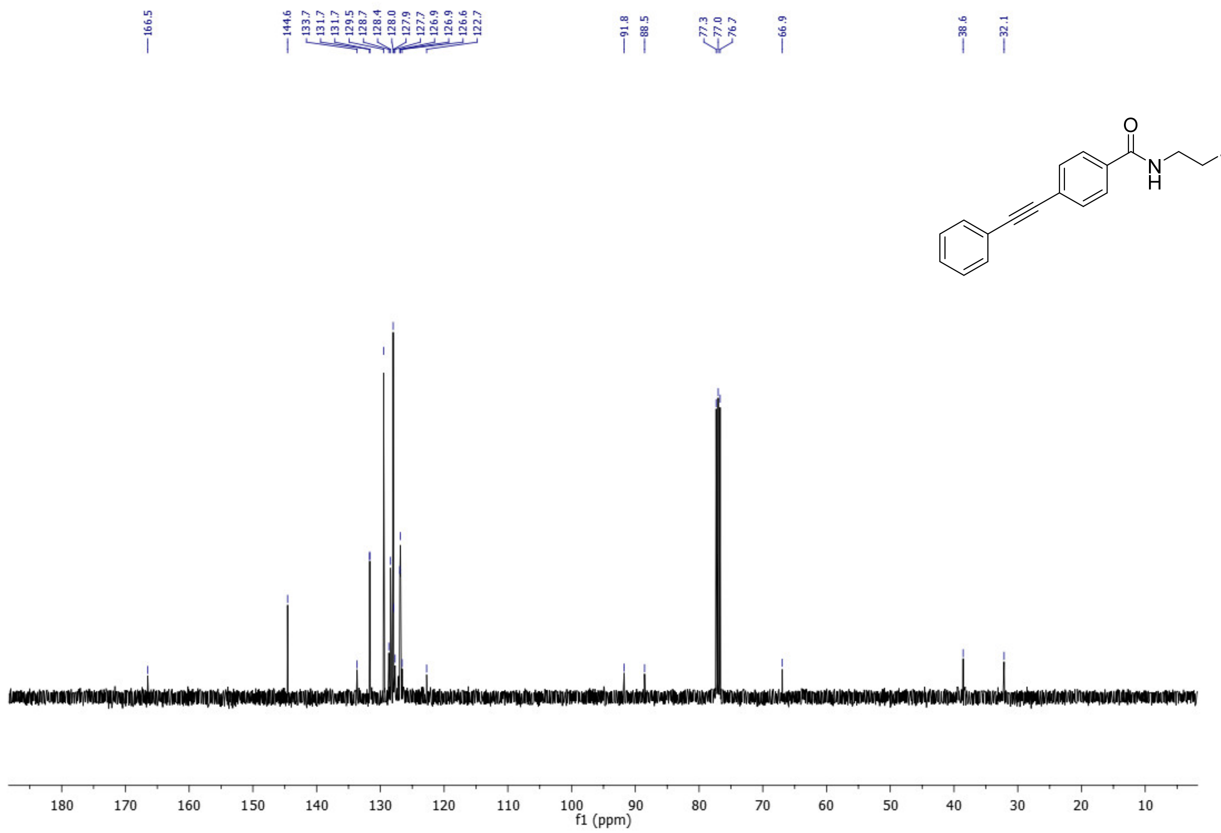

Figure S9. ^{13}C NMR spectrum of compound **3b.**

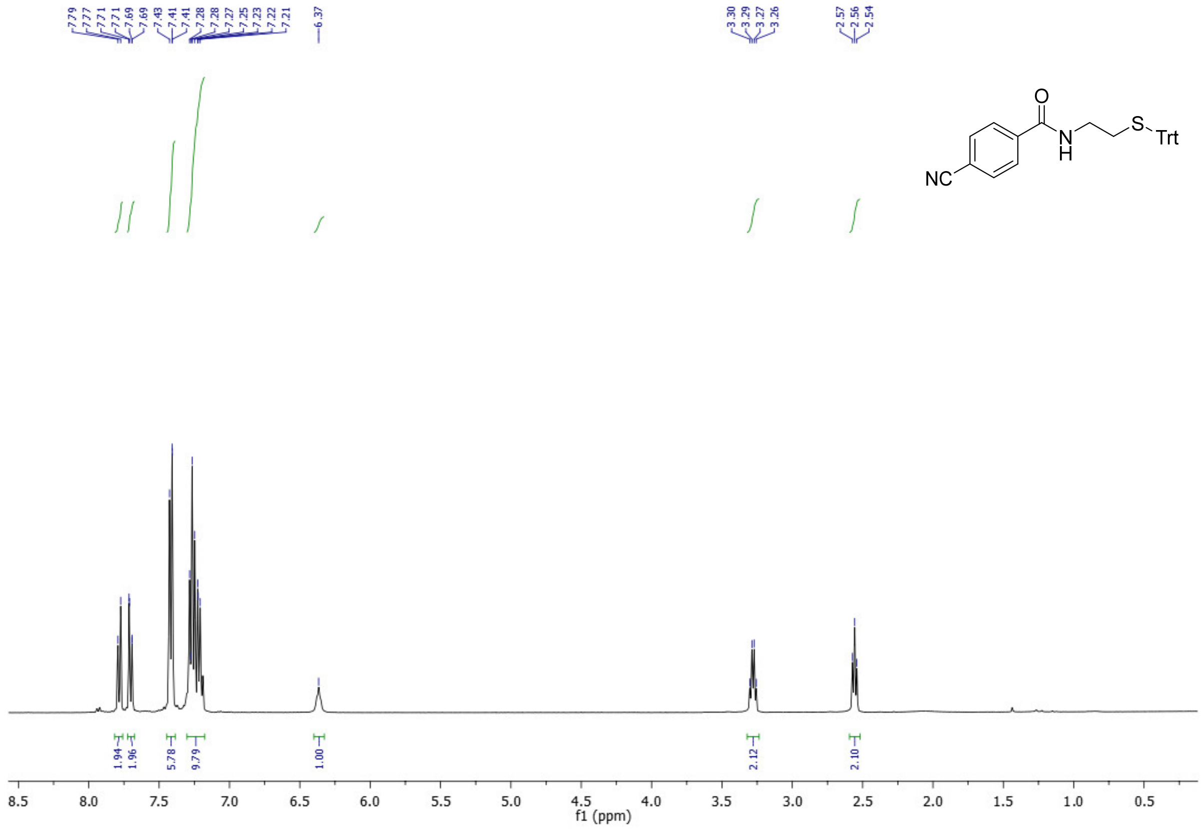

Figure S10. ^{1}H NMR spectrum of compound **3c.**

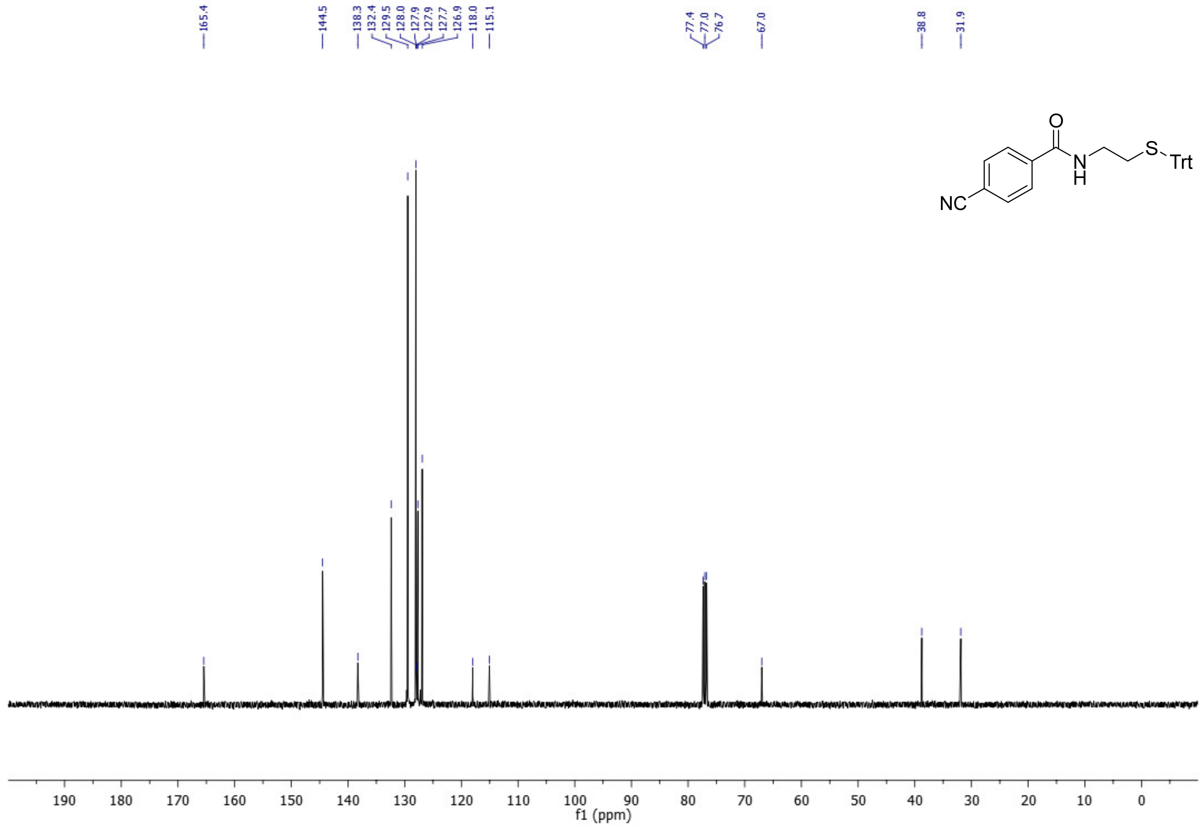

Figure S11. ^{13}C NMR spectrum of compound **3c.**

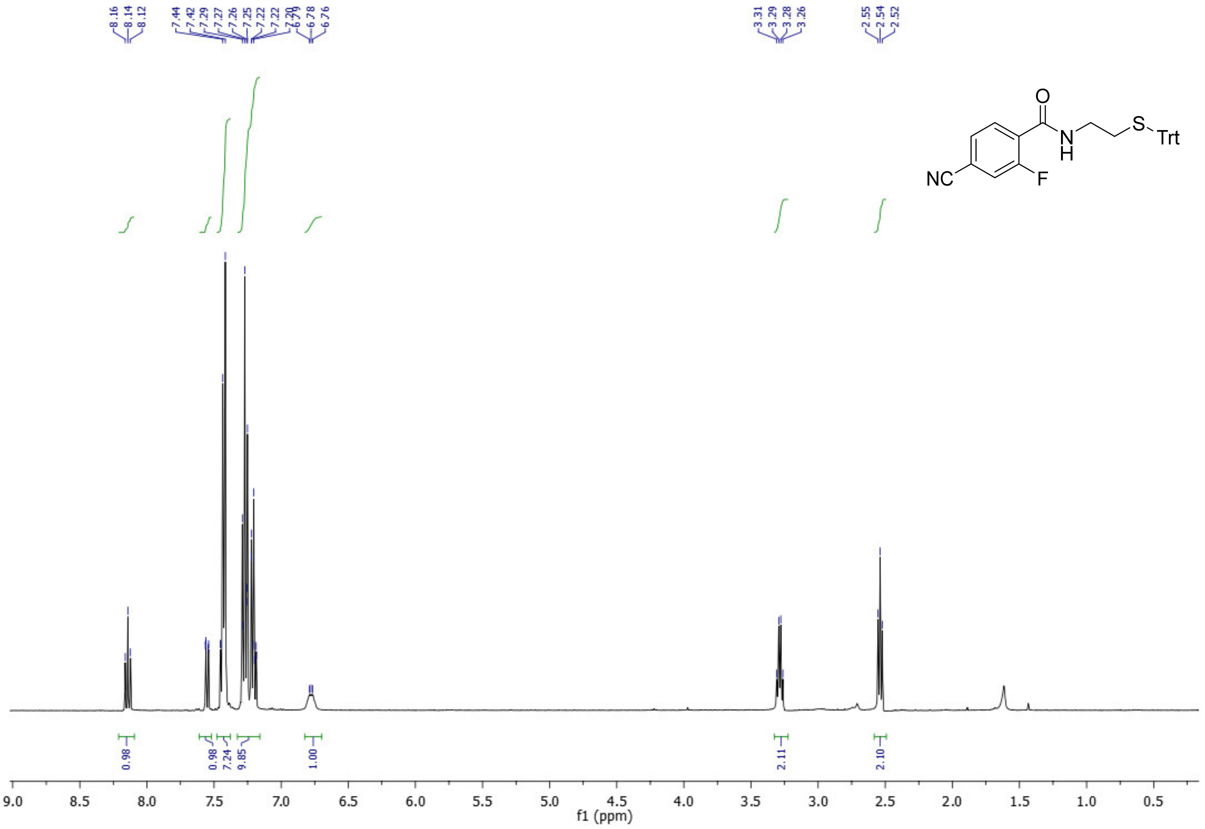

Figure S12. ^{1}H NMR spectrum of compound **3d.**

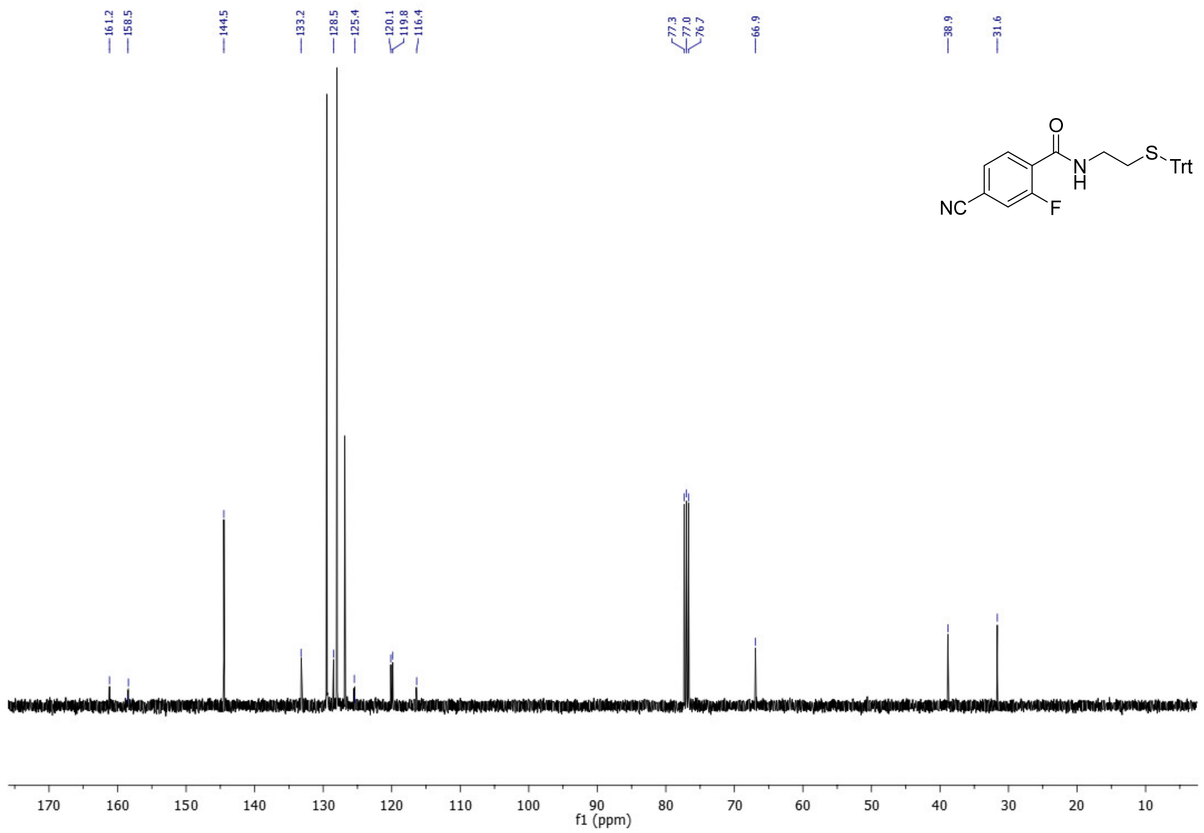

Figure S13. ^{13}C NMR spectrum of compound **3d.**

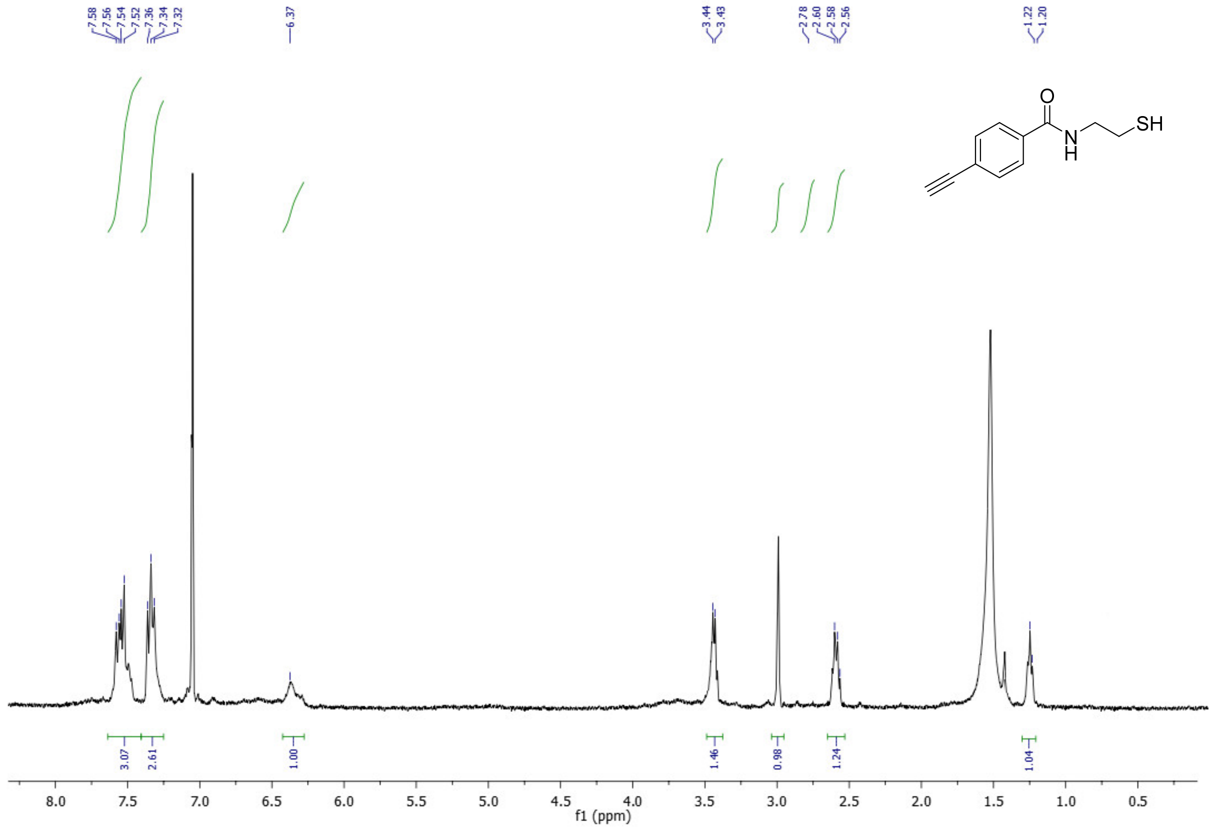

Figure S14. ^{1}H NMR spectrum of compound **4a.**

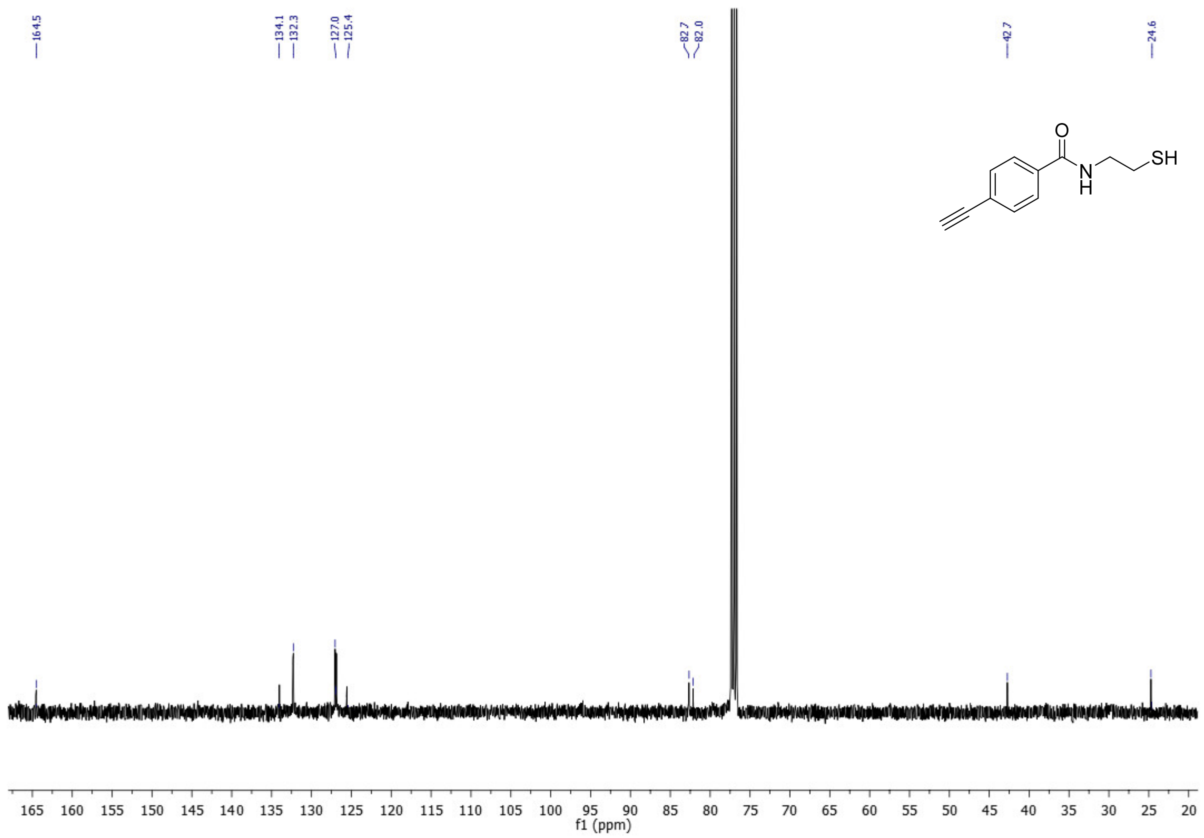

Figure S15. ^{13}C NMR spectrum of compound **4a.**

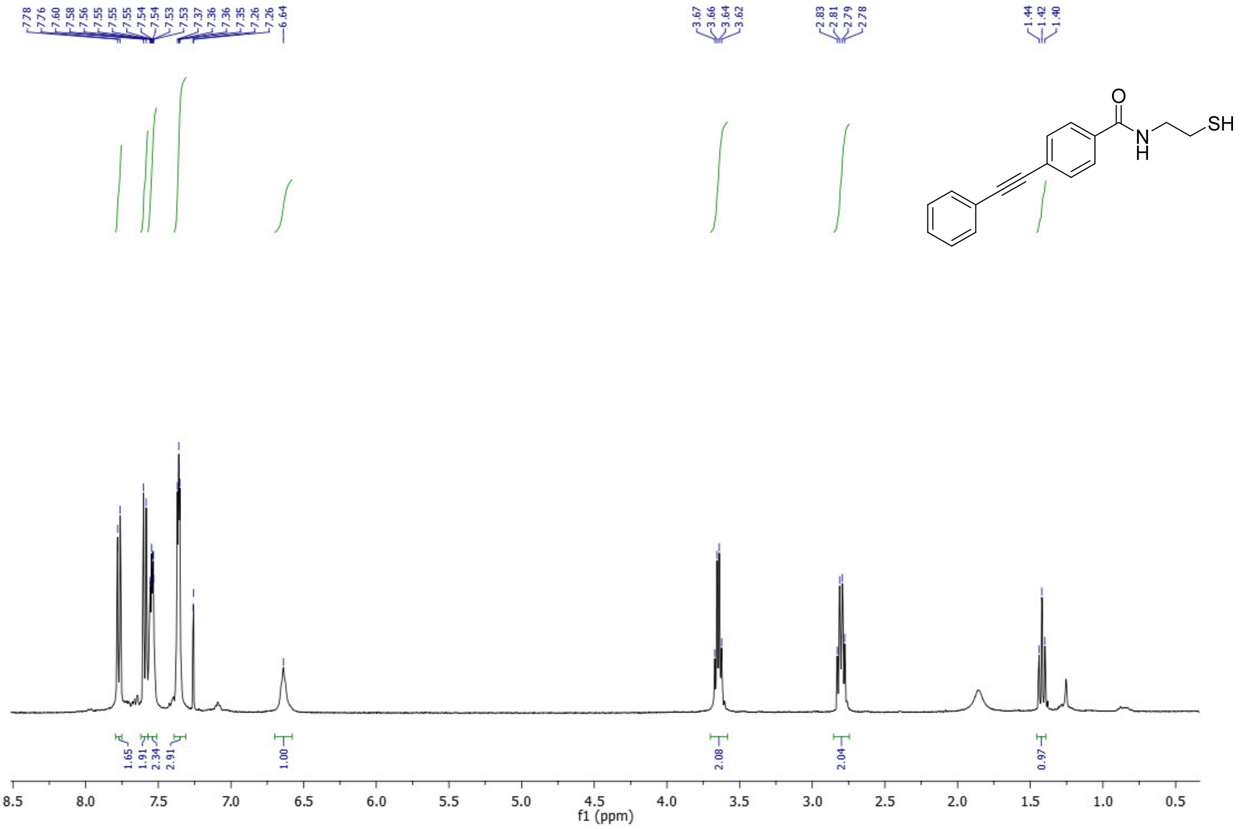

Figure S16. ^{1}H NMR spectrum of compound **4b.**

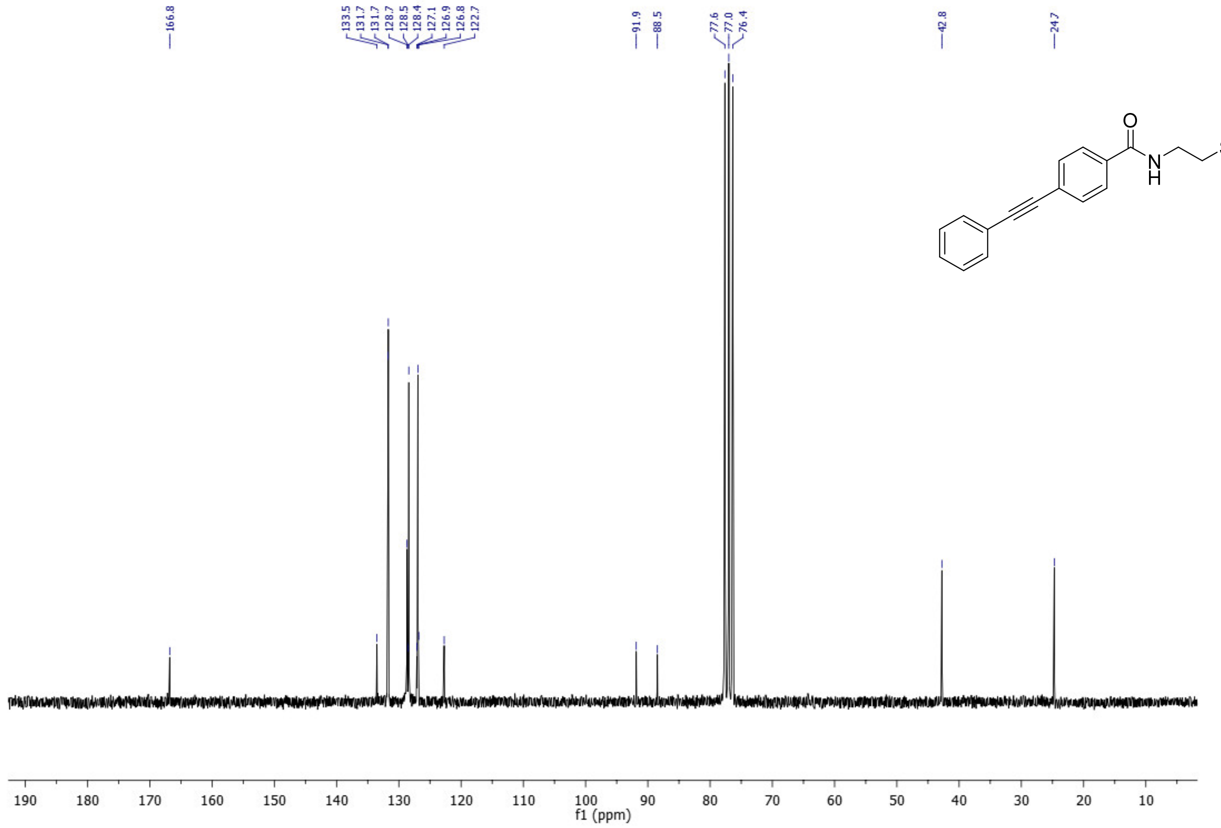

Figure S17. ^{13}C NMR spectrum of compound **4b.**

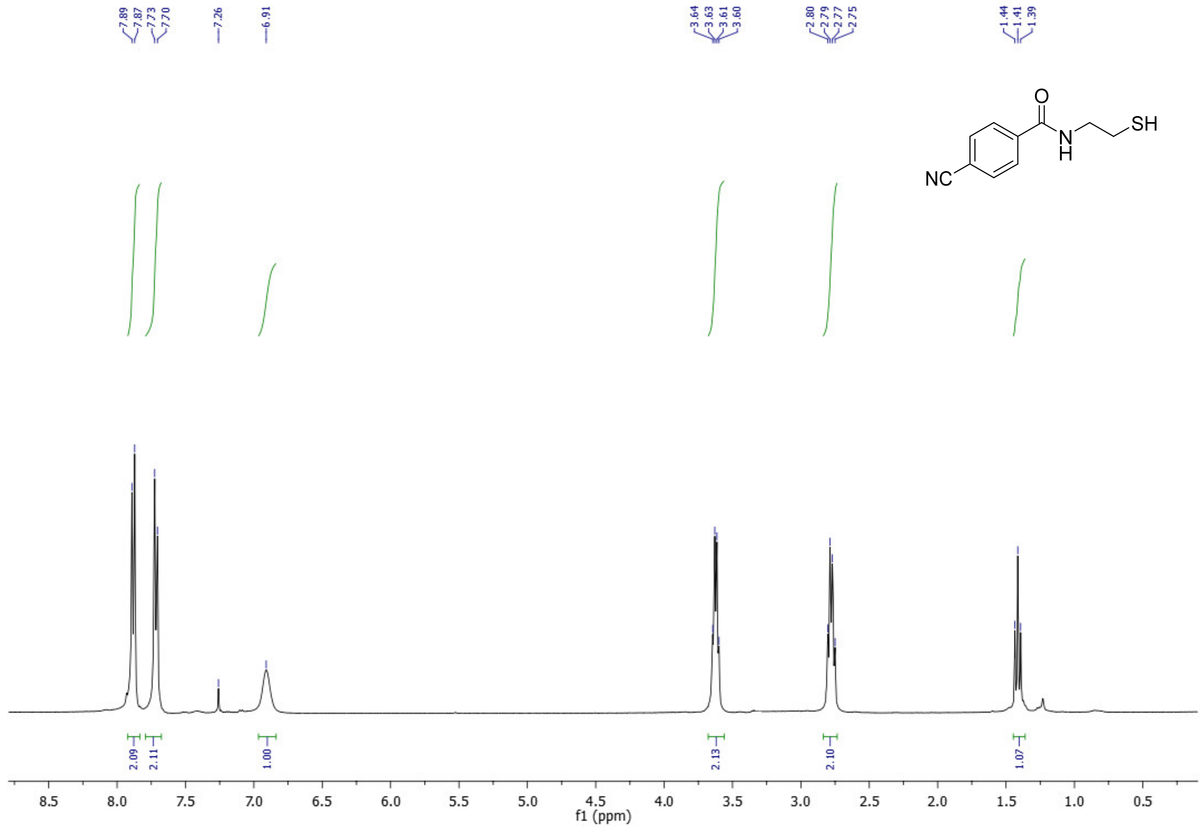

Figure S18. ^{1}H NMR spectrum of compound **4c.**

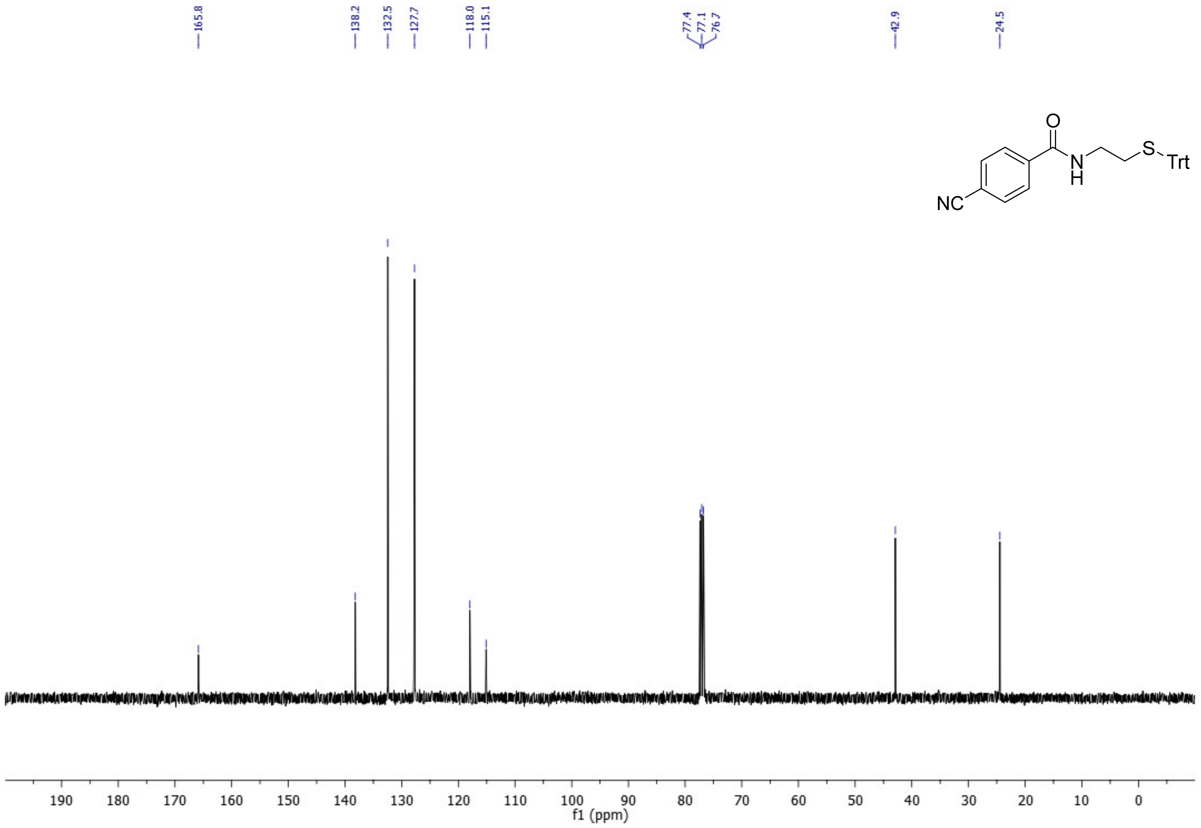

Figure S19. ^{13}C NMR spectrum of compound **4c.**

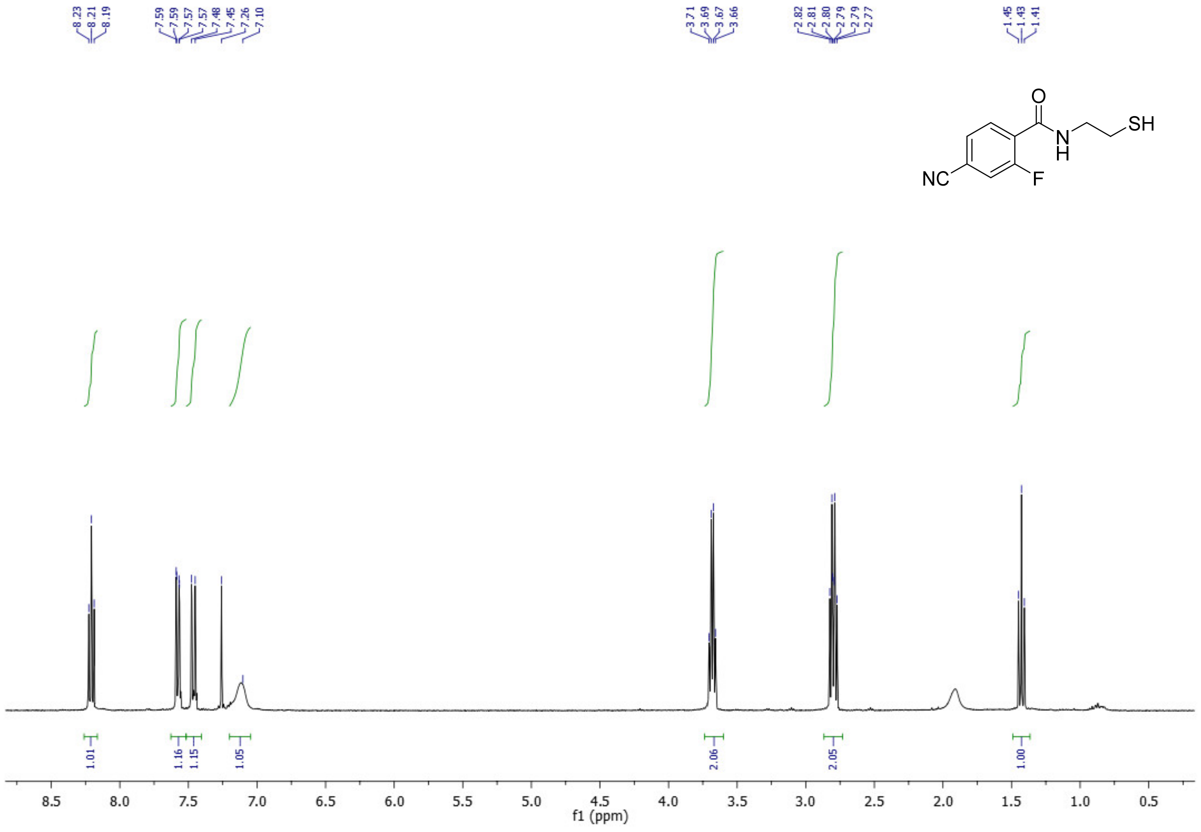

Figure S20. ^{1}H NMR spectrum of compound **4d.**

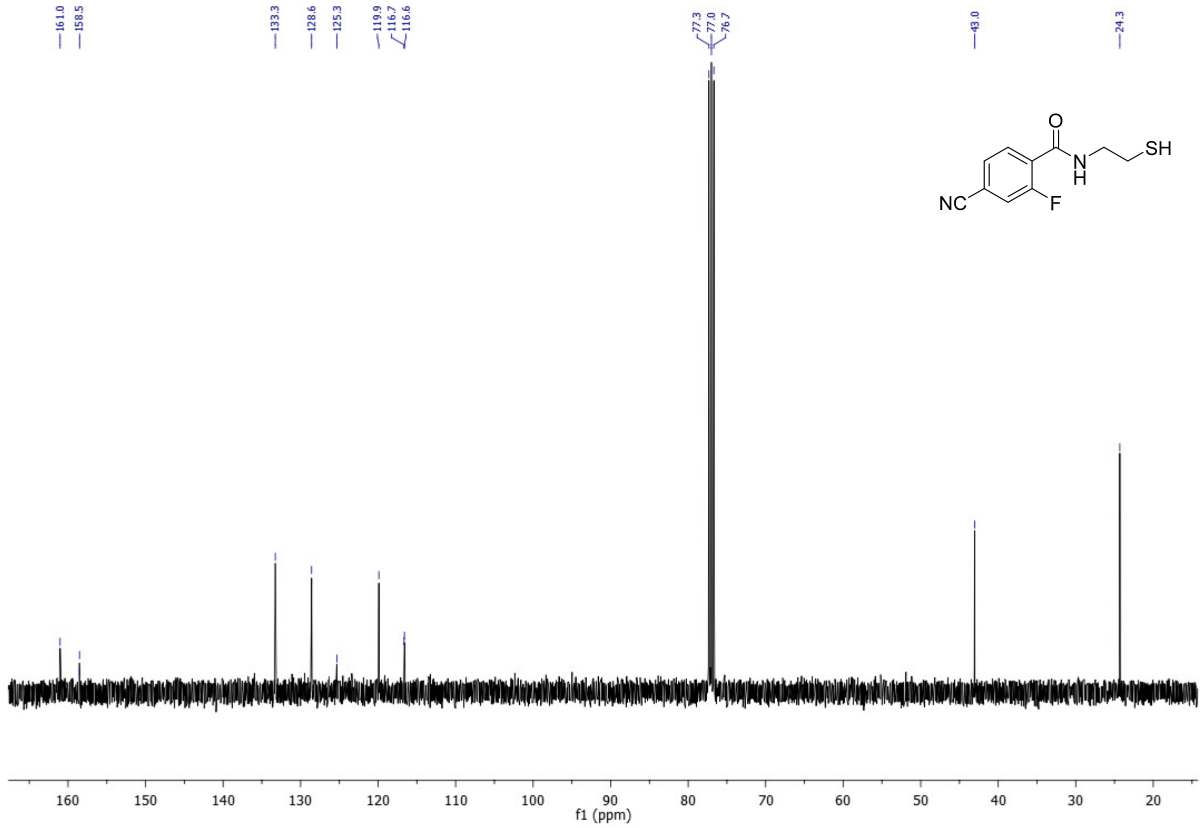

Figure S21. ^{13}C NMR spectrum of compound **4d.**

References

[i] P. A. Dub, R. J. Batrice, J. C. Gordon, B. L. Scott, Y. Minko, J. G. Schmidt, R. F. Williams, *Org. Proc. Res. Dev.* **2020**, *24*, 415.
